# Age-specific effects of density and weather on body condition and birth rates in a large herbivore, the Przewalski’s horse

**DOI:** 10.1007/s00442-023-05477-9

**Published:** 2023-11-16

**Authors:** Heiko G. Rödel, Benjamin Ibler, Katalin Ozogány, Viola Kerekes

**Affiliations:** 1 Laboratoire d’Ethologie Expérimentale et Comparée UR 4443 (LEEC), Université Sorbonne Paris Nord, 93430 Villetaneuse, France; 2 Heimat-Tierpark Olderdissen (Bielefeld Zoo), Dornberger Straße 149a, 33619 Bielefeld, Germany; 3 HUN-REN-UD Behavioural Ecology Research Group, University of Debrecen, Egyetem tér 1, 4032 Debrecen, Hungary; 4 Department of Evolutionary Zoology and Human Biology, University of Debrecen, Egyetem tér 1, 4032 Debrecen, Hungary; 5 Hortobágy National Park Directorate, Sumen utca. 2, 4024 Debrecen, Hungary

**Keywords:** Age-dependence, *Equus ferus przewalskii*, Foaling, Parturition, Precipitation, Reproduction, Senescence, Winter temperature

## Abstract

Reproduction in young females can show a particularly sensitive response to environmental challenges, although empirical support from individual-based long-term studies is scarce. Based on a 20-year data set from a free-roaming Przewalski’s horse population (*Equus ferus przewalskii*), we studied effects of large-herbivore density (horses + cattle) and weather conditions experienced during different life stages on females’ annual birth rates. Foaling probability was very low in 2-year-olds, reaching maximum values in 5 to 10-year-olds, followed by a decrease in older females indicating reproductive senescence. Mother’s previous reproductive investment affected her current reproduction; young and old mothers (as opposed to middle-aged ones), which had nursed a foal for at least 60 days during the previous year, reproduced with a lower probability. Foaling probability and body condition of young females were lower when large-herbivore density was high. Reproduction was also influenced by interactive weather effects during different life stages. Low late-summer precipitation during the females’ year of birth was associated with a pronounced decrease in foaling probability in response to harsh late-winter temperatures prior to the mating season. In turn, increased amounts of late-summer rain during this early age together with more late-summer rain during the females’ current pregnancy led to an increased reproductive probability in 2–3-year-olds. These results were corroborated by the ameliorating effects of late-summer rain on body condition in such females. In conclusion, our findings highlight the interactive importance of weather conditions experienced during early life, and of density and weather during current pregnancy on foaling probability, particularly in young females.

**Supplementary Information:**

The online version contains supplementary material available at 10.1007/s00442-023-05477-9.

## Introduction

Exploring the environmental drivers of age-dependent reproduction is an important step in the study of the dynamics of age-structured populations (Emlen and Pikitch 1989; Coulson et al. 2005; Frederiksen et al. 2014). Typically, high population densities and adverse weather conditions can negatively affect reproductive parameters (Bronson 1985; Fowler 1987). For example, various mammal species show lower birth rates when densities are high (small mammals: Frylestam 1980b; Rödel et al. 2004a; large mammals: Albon et al. 2000; Coulson et al. 2000; Focardi et al. 2000; Stewart et al. 2004; Richard et al. 2014). In particular in herbivores, a key mechanism frequently discussed to underlie such negative effects on fertility parameters is the increased competition for and depletion of food resources at high population densities, negatively impacting the animals’ body condition (cf. Helle and Kauhala 1995; Bonardi et al. 2017). In addition, multiple weather variables have the potential to even concomitantly affect vital rates including reproduction (Rödel et al. 2004b; Louthan et al. 2021), predominantly via their impact on food availability or quality. Studies in different species of ungulates, including feral domestic horses (*Equus ferus caballus*), support negative effects of harsh winter weather on annual birth rates (Coulson et al. 2000; Richard et al. 2014). Such effects can be enhanced when population density and different weather variables operate interactively (Stewart et al. 2004; Rodriguez-Hidalgo et al. 2010; Richard et al. 2014; Gamelon et al. 2017).

At the individual level, reproductive performance frequently changes with age. There is increasing support for reproductive senescence in old females compared to prime-aged (usually middle-aged) mothers from wild or free-ranging populations, for example in terms of reduced litter sizes or lower birth rates in the former (Gaillard et al. 2000; Rödel et al. 2004a; Turbill and Ruf 2010). Furthermore, young or primiparous females often show a comparatively lower (Garrott et al. 1991; Helle and Kauhala 1995; DelGiudice et al. 2007) or at least a more variable reproductive performance than older mothers (review in: Gaillard et al. 2000). Studies in ungulates revealed that such high variation in young females may be attributed to their relatively high sensitivity to challenging environmental conditions, as exemplified by the comparatively stronger decrease in young females’ birth rates in response to high density (bighorn sheep *Ovis canadensis*: Festa-Bianchet et al. 1995, Soay sheep *O. aries* and red deer *Cervus elaphus*: Coulson et al. 2000) or harsh winter weather (moose *Alces alces*: Markgren 1969, red deer: Coulson et al. 2000). In turn, the lower sensitivity of vital rates to environmental variation in prime-aged compared to young females may contribute to buffer the effects of fluctuating environmental conditions on changes in population growth (Morris and Doak 2004; Hilde et al. 2020).

The individual reproductive history is a further parameter potentially influencing the reproductive probability or performance of females. As reproduction, and in particular lactation is energetically costly (McNab 2002; Speakman 2008), a relatively high current reproductive effort can be expected to alter the reproduction or survival of an individual in the future (Stearns 1992). In long-lived species such as in ungulates, which typically follow a slow life history strategy, the costs of reproduction can be expected to mainly be apparent in terms of a lower future reproduction rather than by a reduced future survival of the mother (Hamel et al. 2010a). Even if the exact physiological mechanisms driving these life-history trade-offs are still poorly understood, it has been suggested that the costs of reproduction are especially evident when females are exposed to unexpected and unfavorable environmental conditions (Zhang and Hood 2016). Furthermore, some studies in ungulates indicate that reproductive trade-offs can be particularly pronounced in young, primiparous mothers, as evident by their reduced reproductive probability during the following year (feral horse *Equus caballus*: Garrott and Taylor 1990; Soay sheep *Ovies aris*: Regan et al. 2022).

Furthermore, challenging conditions experienced during early life can have long-lasting effects on individual life histories including on reproductive traits (Descamps et al. 2008; Rödel et al. 2009). For example, female Soay sheep experiencing high population densities during the summer after birth showed a lower probability of reproduction as yearlings (Forchhammer et al. 2001), and a similar delayed effect of early-life population density on the probability of pregnancy later in life was found in a Mediterranean red deer population (Rodriguez-Hidalgo et al. 2010).

In summary, different environmental interactions can affect parameters of reproduction on the short-term as well as on the long-term, and features such as age can alter an individuals’ susceptibility to such effects. However, integrative studies taking into account the interplay of these various parameters are scarce, as the necessary individual-based long-term data are difficult to collect and thus rarely available (review in: Schradin and Hayes 2017).

We analyzed such a long-term data set collected from a population of a large mammal, the Przewalski’s horse (*Equus ferus przewalskii*), living under natural conditions in a 3000-hectare area at the Hortobágy National Park in Hungary (Kerekes et al. 2019, 2021). Our main goal was to investigate the interactive effects of different weather parameters and density as well as the impact of the individual reproductive effort during the previous year on the females’ age-specific foaling probability during the annual breeding season. The Przewalski’s horse is a particularly interesting model for the study of harsh weather conditions, as its natural semi-arid steppe habitat can provide challenging environmental conditions by hot summers with poor vegetational quality as well as by cold winters (Janssen et al. 2016). Przewalski’s horses, which are considered as the last truly wild horses, diverged from ancestors of the domestic horse (*E. f. caballus*) around 45,000 years ago (Der Sarkissian et al. 2015), and are listed as ‘Endangered’ in the international IUCN Red List of Threatened Species (King et al. 2015). Przewalski’s horses are seasonal breeders and the majority of the foals is usually born in May and June (Chen et al. 2008). Similar to the domestic horse (Heck et al. 2017), the gestation period of the Przewalski’s horse is around 330–340 days (Monfort et al. 1991; Maltzan et al. 2007), and mothers usually give birth to a single foal (Chen et al. 2008).

The study area in the national park included, next to the Przewalski’s horses, a herd of semi-wild cattle (reconstructed aurochs, *Bos taurus taurus*; Kerekes et al. 2019). In years with increased densities of these two large grazers, the ground vegetation, i.e., the horses’ only food source, was increasingly depleted (Kerekes et al. 2019). Thus, we (i) predicted that following such high-density years, possibly either due to a decreased probability of successful conceptions or by increased (early) pregnancy losses (Satué and Gardon 2016), the probability of giving birth during the next foaling season will be lower. Furthermore, (ii) low winter temperatures, in particular during the late winter season, which have the potential to delay the green-up of the grass vegetation (cf. Mech et al. 1987; Rödel et al. 2005), might lead to a decreased rate of successful conceptions during the following months. As a consequence, low winter temperatures may negatively affect females’ foaling probability with a delay of 1 year (corresponding to Przewalski’s horses’ gestation time of 11–12 months). In contrast, (iii) high amounts of precipitation during late summer, thus counteracting the negative effects of the usual summer droughts and leading to the regrowth of green pasture prior to the winter season, might increase the animals’ body condition and thus may decrease the probability of pregnancy losses (Satué and Gardon 2016). Therefore, we predicted that rains in late summer may have increased the probability that females will give birth during the following year. Most importantly, (iv) we focused on age-specific responses to such environmental challenges, as young horses (see Gaillard et al. 2000 for a review on other ungulates) may be particularly sensitive to such effects. That is, we predicted that birth rates in young females would be prone to more pronounced negative density effects and show stronger responses to harsh winter conditions. Younger females, supposedly in a lower body condition, may also respond particularly sensitively to the absence of late-summer rains, and thus such conditions may lower the foaling probability in this age class during the following season. We (v) also explored possible long-term consequences of late-summer rains to which foals were exposed to during their first year of life on their later reproductive performance. We predicted that foals experiencing more advantageous early life conditions in terms of more late-summer rain, thus potentially benefitting from higher food quality, would show higher birth rates during early adult stage. Finally, we (vi) predicted that possible direct (year-to-year) costs of reproduction in terms of reduced future reproduction, i.e., a lowered foaling probability of females which had already reproduced during the previous year, should be particularly pronounced in young females. Based on a smaller sample size from 6 years, we (vii) also explored potential effects of these different environmental challenges on female body condition, as such effects may provide some insights into the mechanisms linking weather and density to female reproductive performance (Frylestam 1980a; Rödel et al. 2005; Flajšman et al. 2017).

## Materials and methods

### Study population

The study was conducted on animals from a Przewalski’s horse population living in a fenced area of 3000 ha (2400 ha before 2018) in the Pentezug reserve of the Hortobágy National Park in Hungary. The area is an alkali grassland with marshes and few interspersed groups of trees along a river (Kerekes et al. 2019). The natural grass pasture was the animals’ only food source. The first horses were introduced to the Hortobágy National Park in 1997, and since then the population size increased (Kerekes et al. 2021). The Pentezug reserve was (and still is, in the year of publication) also inhabited by another large grazer, a herd of semi-wild cattle (reconstructed aurochs), which were also reproducing and growing in population size since their introduction in 1999 (Kerekes et al. 2021). There were no large predators in the area. No visitors were allowed in this part of the national park, although the horses were approached and observed by staff of the national park for population survey at an almost daily basis.

Furthermore, for a necessary population control, several of the female horses were injected with an immunocontraceptive (porcine zona pellucida, PZP) during the years 2013–2015 and 2017–2019, with around 15 females treated per year (more details in: Kerekes et al. 2021). The treatment related to the PZP injection procedure was minimum invasive, since animals were not captured, but the injections were administered from a distance using a blowgun. During the different years of treatment, different selection criteria were applied, as either younger or older females were preferably chosen. Most importantly, starting with the year a female was subjected to this PZP treatment, its data were excluded from the analysis presented in this paper (see details in Table A in Suppl. Materials). Nevertheless, (a) for five of our focal females for which reproductive probabilities were assessed, mothers were treated with PZP when these females were still at the foal stage. (b) For another five of our focal females, mothers were treated with PZP prior to their pregnancy with those females, i.e., the PZP treatment was obviously not always efficient in preventing pregnancies. However, our analyses revealed that the age-dependent reproductive probabilities of these females with PZP-treated mothers of conditions (a) and (b) did not differ significantly from females of untreated mothers (LMM: (a) ${\chi }_{1}^{2}$ = 0.001, *p* = 0.988; (b) ${\chi }_{1}^{2}$ = 0.011, *p* = 0.916), and thus these 10 females were kept in our data set for further analyses.

### Reproductive activity of females

#### Study period and sample size

We explored the females’ annual probability of parturition (events at the individual level; foaling: yes/no) starting at an age of 2 years, when Przewalski’s horse females potentially start to reproduce (Kerekes et al. 2021). We only considered females born inside the Pentezug reserve. In total, data for this analysis were collected during 20 years (2000–2019), from a total of 146 females, born between 1998 and 2017. Females were between 2 and 16 years old when their reproductive activity was surveyed. This resulted in a sample size of *n* = 712 observations of presence (57.4%, 409/712) or absence (42.6%, 303/712) of annual parturitions.

#### Surveys of reproductive activity

The population was surveyed for the occurrence of new foals in all harem groups (i.e., one stallion with up to ten adult females including their offspring; see: Klimov 1988; Kerekes et al. 2021; Ozogány et al. 2023), around 5–6 times a week during the main foaling season (late April to late May), and around 2–3 times a week during the rest of the year, when parturitions were less frequent (Volf 1996). This was done by two to three-hour checks by car. Harem groups with present foals, which could be easily spotted from the distance by the aid of binoculars, were approached closely to around 50 m. Mothers were assigned by the nursing of their foal. Horses of this population were not individually marked, although all adult females—including the mothers—could be identified by the trained personnel based on the combination of different characteristics and patterns such as differences in coloration, stripes on the legs, shoulder crosses etc. (see a detailed description of individual identification in: Kerekes et al. 2021). Assignments of mothers based on observational data were re-confirmed by genetic maternity assessments in around 90% of cases (Kerekes et al. 2021). These genetic analyses revealed that only 2.5% of observational assignments of mothers were inaccurate, and these were corrected later on in our data base.

#### Mothers’ previous reproductive effort

**Fig. 1:**
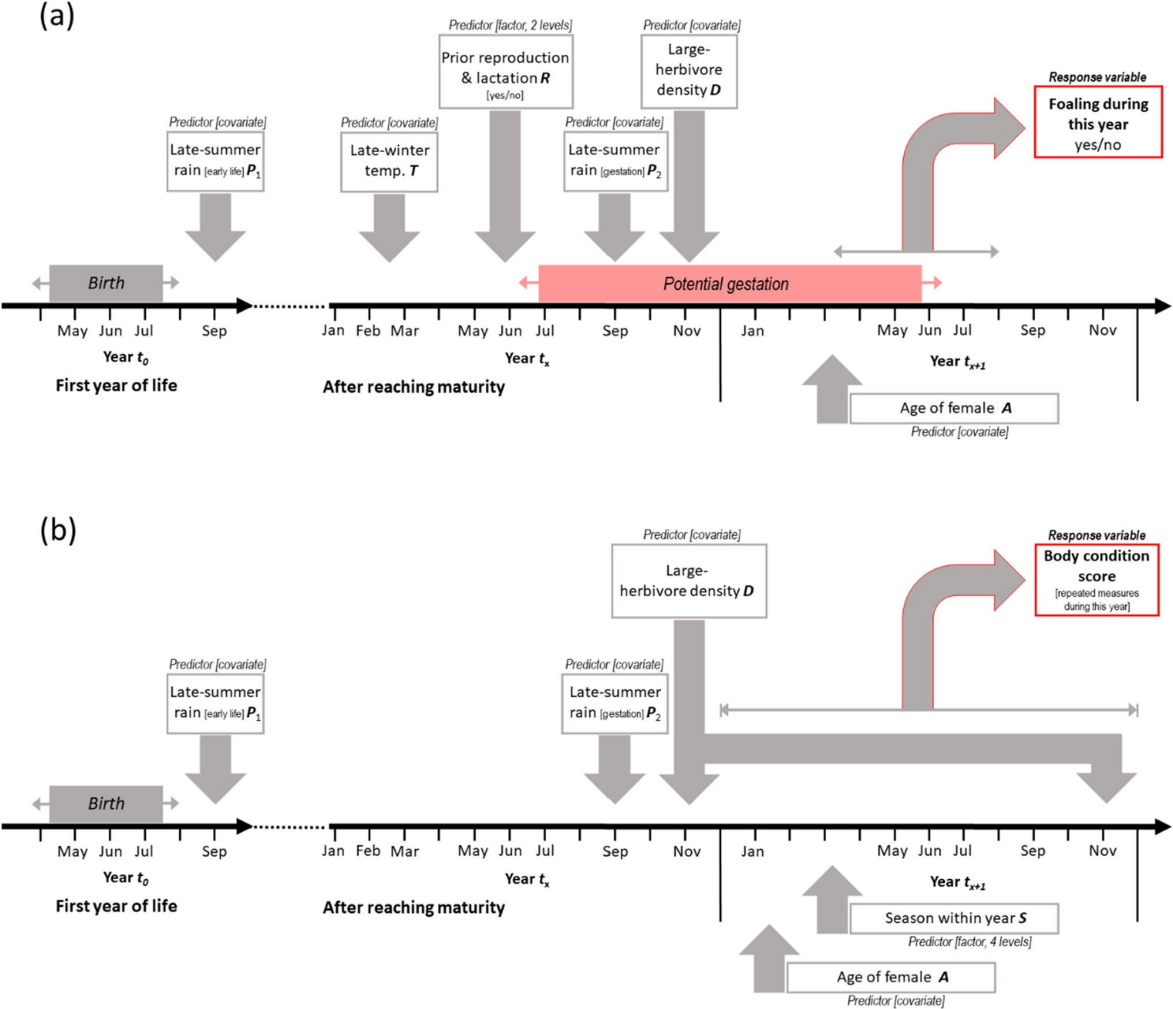
Schemas of the different variables used for multifactorial statistical analyses. **a** Outline of analyses related to foaling probabilities (Table 1 and Table A in Suppl. Materials). The effects of prior reproductive investment (*R*) were explored by a separate analysis, only including females of at least 3 years. **b** Outline of the analysis related to body condition scores (Table 2). For analysis, large-herbivore density (*D*) in association with body condition scores collected in winter (January–April) was quantified in November/December of the previous year (*t*_x_) whilst D in association with scores collected during the remaining year was quantified in November/December of the current year (*t*_x+1_)

We assessed mothers’ reproductive effort during the previous year (***R***), which was used as a factor with two levels in our multifactorial analysis (see details below and in Fig. 1a). Therefore, by our regular surveys (see above), we determined the survival of foals until postpartum day 60, until the time when in the domestic horse approximately the peak in lactation is reached (Oftedal et al. 1983). Note that in our population, the peak foal mortality during the first year occurred during early postnatal life (around postnatal day 17, median). The vast majority, more than 90% of the foals surviving until postnatal day 60 were still with their mother during the following spring.

Lactation imposes considerable energetic costs, as in larger mammals the energy expenditure of lactating mothers is at least 1.5 times higher than of non-lactating ones (McNab 2002). Compared to the high energetic costs of lactation, gestation is usually considered to be less costly, as exemplified by studies showing that the differences in energy demands between non-breeding and pregnant females can be rather low (Gittleman and Thompson 1988; Speakman 2008; Rödel et al. 2016). Thus, for later analysis, and in accordance to a study in Soay sheep (Regan et al. 2022), we distinguished between females which (a) had not reproduced during the previous year or whose foal had died shortly after parturition, within the first 60 days, and (b) females which had reproduced during the previous year and whose foal was alive at least until postnatal day 60 (see more details below and in results). Note that in our study in the Pentezug reserve, mares exclusively gave birth to singletons.

### Body condition scores of females

We also analyzed weather and density effects on females’ body condition based on a data set collected during 6 years; see below for details on sample sizes and a schema in Fig. 1b.

Following the method developed by Rudman and Keiper (1991) for feral ponies (*E. ferus caballus*), we used a body condition score from 0 (very low body condition, although animals with such a low score were never observed during our study) to 5 (very high body condition), in steps of 0.5. As the basis of this score, we assessed the shape of the horses’ hind quarters from an observer position behind the animal, by the aid of binoculars, from a distance of around 20 to 40 m. Detailed drawings describing the method of quantification can be found in Rudman and Keiper (1991), and more details on the application of this method to Przewalski’s horses are in Kerekes et al. (2019).

### Density of large herbivores (horses and cattle)

The number of cattle in the Pentezug reserve was counted once per year, in November or December when all cattle were locked into a small and closed area for veterinary inspection. The number of horses was known due to regular surveys at the individual level; that is, individual compositions of all harem groups were known (see above in “Surveys of reproductive activity”). Foal mortalities and their survival times were known due to surveys at the daily to weekly basis (more details in: Kerekes et al. 2021). For consistency with density data available from cattle, we used the density of horses assessed in November/December of each year. During the study period, November/December densities of horses varied from 0.08 to 1.37 individuals per 10 ha, and densities of cattle varied from 0.05 to 2.41 individuals per 10 ha. In the beginning of the study period (in 2000), the densities of horses and cattle were 0.08 and 0.05 individuals per 10 ha, and in the end of the study period (in 2019), the densities were 0.93 and 0.64 individuals per 10 ha, respectively. Annual densities of horses and cattle were strongly and positively correlated (*R*^2^ = 0.913, *β* = 0.956 ± 0.069 SE, *p* < 0.001). Changes in densities of horses and cattle were due to variation in reproduction and survival. Furthermore, each year, several cattle were transported into or out of the study area. To a minor extent, this was also true for the horses, as some individuals were transferred from or to other enclosure populations (e.g., to zoological parks). However, note that all Przewalski's horse females, which were not born in our study population were excluded from the analysis of foaling probabilities.

***D***: For statistical analysis, we used the total density of cattle and horses (all age classes of both species per area size), hereafter referred to as ‘large herbivore density’ (see Fig. 1a, b).

### Weather data

Data on precipitation (daily amounts) and on ambient temperatures (daily averages) were obtained from a close-by meteorological station (Debrecen, 38 km away from the center of the Pentezug reserve), situated at the same altitude of around 120 m a.s.l.. Based on these daily values, we calculated different weather variables, used as predictor variables for our statistical analyses.

***P***_***1***_: The summed-up amount of precipitation in late summer (September) during the females’ year of birth (see Fig. 1a, b). Observations (by VK and colleagues, unpublished) during the last decades had revealed that rainy weather in late summer leads to a notable regrowth of green pasture, thus increasing the quality and quantity of the horses’ only food source. As stated upfront, we predicted that such a boost in food availability during early life may positively affect the body condition of the foals with potential long-term effects on their reproductive performance (Lummaa and Clutton-Brock 2002). In few cases, females were either born during September (7 out of 146 = 4.8%) or were born shortly after, in October (6 out of 146 = 4.1%). We did not exclude these cases from our analysis of reproductive probabilities, as such late-born foals might have at least benefitted indirectly from higher amounts of late summer precipitation, possibly via mother’s higher energy intake and thus increased lactational performance. However, when excluding these cases of late-born foals (*n* = 13 females) from statistical analyses, we obtained the same results.

***P***_***2***_: The summed-up amount of precipitation in late summer (September) during the females’ potential pregnancy (see Fig. 1a, b). We predicted that the regrowth of green pasture related to higher amounts of rain during this period will improve the chance that females will keep their (potential) pregnancy during the critical early period of gestation (cf. domestic horse: Satué and Gardon 2016).

***T***: The average ambient temperature during late winter (February/March) for each year of the study period. For analysis of females’ foaling probability, this variable was calculated for late winter prior to the female’s breeding season, i.e., 1 year prior to the potential parturition in focus. This time-delayed effect of winter temperature was taken into account since we predicted that harsh winter conditions may have possibly decreased the chance of a successful conception thereafter (see Fig. 1a).

### Statistical analysis

#### Effects of different predictors on foaling probability

Statistical analyses were done with the program R, version 4.3.0 (R Core Team 2023). We analyzed the effects of different predictor variables on females’ probability of foaling (binary response variable) by generalized linear mixed-effects models (GLMM) for binomial data with a logit link, fitted by the Laplacian maximum likelihood approximation, using the R package *lme4* (Bates et al. 2015). *P*-values were calculated by type-3 Wald chi-square tests (Bolker et al. 2009).

This analysis was based on a total of 712 observations (occurrence of annual foaling: yes/no) from 146 females (2–16 years old) over a period of 20 years. We included female identity as a random (intercept) factor, since the data set included repeated measurements of individual females across consecutive years. The current year of reproduction was included as a further random factor to adjust for year-to-year variation which remained unexplained by the environmental predictors considered.

These environmental predictor variables (all covariates), which are defined above, were *D*, *P*_1_, *P*_2_ and *T* (details in Fig. 1). We also considered the females’ age in years (*A*, covariate), since in various species of mammals and birds, young adult females often show a lower reproductive performance compared to older age classes (Clutton-Brock 1988; Rödel et al. 2004a; Monclús et al. 2014). In addition, we tested for polynomial (quadratic) effects of age on foaling probability, predicting an initial increase in reproductive performance with a maximum in middle-aged females, followed by a decrease in old females, as it has been described in several studies on small and large mammals (Rödel et al. 2004a; Hayward et al. 2013; Nussey et al. 2013). All covariates were scaled for analysis, i.e., they were centered to the mean which was set to zero and the standard deviation to 1.

As a further predictor variable (2-level factor), we considered the females’ previous reproductive effort (*R*); see details above. As Przewalski’s horses do not give birth before reaching at least an age of 2 years (Kerekes et al. 2021, and details in “Results” section), the inclusion of this age class would lead to a strong collinearity between age and previous reproductive effort. Thus, we ran the analysis including this factor on a reduced data set by removing all data from 2-year-old females, leading to a sample size of *n* = 566 observations from 107 females. However, we still analyzed the effects of previous reproductive effort by a multifactorial model, including the same set of predictors as described above (see Table B in Suppl. Materials).

We tested all 2-way interactions between the predictors considered (see Table 1 and Table B in Suppl Materials). Non-significant interactions (*p* > 0.05) were stepwise removed and models were re-calculated (Engqvist 2005). We visually checked for temporal autocorrelations by plotting the years of study versus the model residuals, revealing a random pattern without any indications for autocorrelations. We also checked for the occurrence of (multi)collinearities using variance inflation factors (VIF). This was done for all models, including all interactions. As VIF were always lower than 3.5, there were no indications of interfering (multi)collinearities (Faraway 2006).

**Table 1:** Effects of different predictor variables on the age-specific foaling probability of female Przewalski’s horses

Source of variation	*χ*^2^	*df*	*Estimates* ± *SE*	*p*
Female age (2nd order polynomial effect) *A*	117.528	2	− 25.999 ± 3.740	**< 0.001**
Large grazer density after potential conception *D*	22.269	1	− 0.918 ± 0.195	**< 0.001**
Precipitation in late summer during 1st year of life *P*_1_	1.911	1	0.160 ± 0.116	0.167
Precipitation in late summer after potential conception *P*_2_	1.281	1	0.212 ± 0.187	0.258
Temperature in late winter prior to potential conception *T*	0.376	1	0.118 ± 0.193	0.540
*D* × *A*	4.239	1	0.315 ± 0.153	**0.034**
*P*_1_ × *A*	0.006	1	− 0.011 ± 0.147	0.941
*P*_2_ × *A*	0.096	1	0.046 ± 0.148	0.756
*T* × *A*	0.103	1	− 0.054 ± 0.168	0.749
*P*_1_ × *P*_2_	8.011	1	0.440 ± 0.155	**0.005**
*P*_1_ × *T*	6.128	1	− 0.292 ± 0.118	**0.013**
*P*_2_ × *T*	0.240	1	0.138 ± 0.281	0.624
*P*_1_ × *D*	1.532	1	− 0.151 ± 0.122	0.216
*P*_2_ × *D*	1.820	1	− 0.392 ± 0.201	0.177
*T* × *D*	0.242	1	0.093 ± 0.188	0.623

Analysis by GLMM for binomial data based on *n* = 712 observations from 146 females over 20 years. Non-significant interactions (*p* > 0.05) were stepwise removed and the models were re-calculated. The proportional variance explained by the final model excluding all non-significant interactions was _marginal_ *R*^2^ = 0.532

Significant effects are highlighted in bold

Female identity and current year were used as random intercept factors

#### Effects of different predictors on body condition

The effects of the environmental variables *D*, *P*_1_, and *P*_2_ on female body condition scores (response variable) were tested by multifactorial linear mixed effects models (LMM) using the R package *lme4* (Bates et al. 2015) (Fig. 1b). As the distribution of these equidistant body condition scores (Rudman and Keiper 1991) was unknown, we calculated *p*-values by parametric bootstrapping (1000 iterations) using the package *afex* (Singmann et al. 2022).

The available data set included *n* = 393 measurements of score values from 64 females (2–17 years old) collected all year round during 6 years (2004–2007, 2018, 2019). Thus, the analysis was based on 6 different measurements of *P*_2_ stemming from 6 years, although from 19 different measurements of *P*_1_ (late-summer rain during first year of life), since the females included in this analysis were part of 19 annual birth cohorts. As for each year, only one census of large herbivore density taken in Nov/Dec was available, body condition scores measured during the early season (Jan–Mar) were set in association with the density from the previous year (i.e., with the value of the density census done shortly before), whereas scores measured from April to December were set in association with the density census of the current year, which included the summed-up numbers of foals and calves that survived during that year. In this way, eight different large-herbivore density measurements were included in our analysis. Further predictors considered in this analysis were females’ age in years (*A*, covariate) and the season (*S*) during which the measurements were taken (factor with 4 levels; Jan–Mar; Apr–Jun, Jul–Sep, Oct–Dec) (Fig. 1b). Covariates were scaled for analysis (details above), and female identity and year were used as random (intercept) factors.

We did not include the effects of late winter temperature (*T*) in this analysis, as body condition scores from April on, i.e., after the end of this late-winter period, were only available from 4 years. Thus, the sample size (*n* = 4 years) was too low to allow such a correlative analysis between *T* and body condition scores.

When analyzing the effects of *P*_1_ on body condition, we did not only test for linear but also for (second-order) polynomial effects. Even if increasing precipitation levels during late-summer rain may improve the animals’ body condition via the green-up of the ground vegetation, in particular during foal stage, extremely high amounts of rain may have negative consequences, leading to a non-linear (inverted U-shaped) association. Such potentially negative effects on body condition and survival may be mediated via a higher persistence of infective stages of endoparasites outside the host’s body when the ground vegetation is consistently wet (e.g., Rödel and Starkloff 2014; Bond et al. 2023).

We tested all 2-way interactions between female age and the environmental variables *D*, *P*_1_, and *P*_2_*.* Further interactions among the environmental variables were not considered due to the rather moderate number of different years available for this analysis. Variance inflation factors were always lower than 3.6, thus models showed no indications of interfering (multi)collinearities (Faraway 2006).

## Results

### Reproduction

Out of the 146 females included in our study, 85 (58.2%) gave birth at least one time during the 20 years considered for analysis (2000–2019). Parturitions occurred from early February to late November. The vast majority, 80% of these took place in spring/summer (*10th percentile*: April 29th, *90th percentile*: July 17th), with an average parturition date on May 30th (median parturition date: May 19th).

#### Age effects

The onset of reproductive activity was age-dependent, with the majority of females starting to give birth when 3 years old. In detail, 3.4% of mothers (5 out of 146) started to give birth at an age of 2 years, 56.1% (60 out of 107) at an age of 3 years, 17.4% (16 out of 92) at an age of 4 years, and 5.1% (4 out of 78) of the females started when 5 years old. None of the females included in our study reproduced for the first time when older than 5 years.

**Fig. 2:**
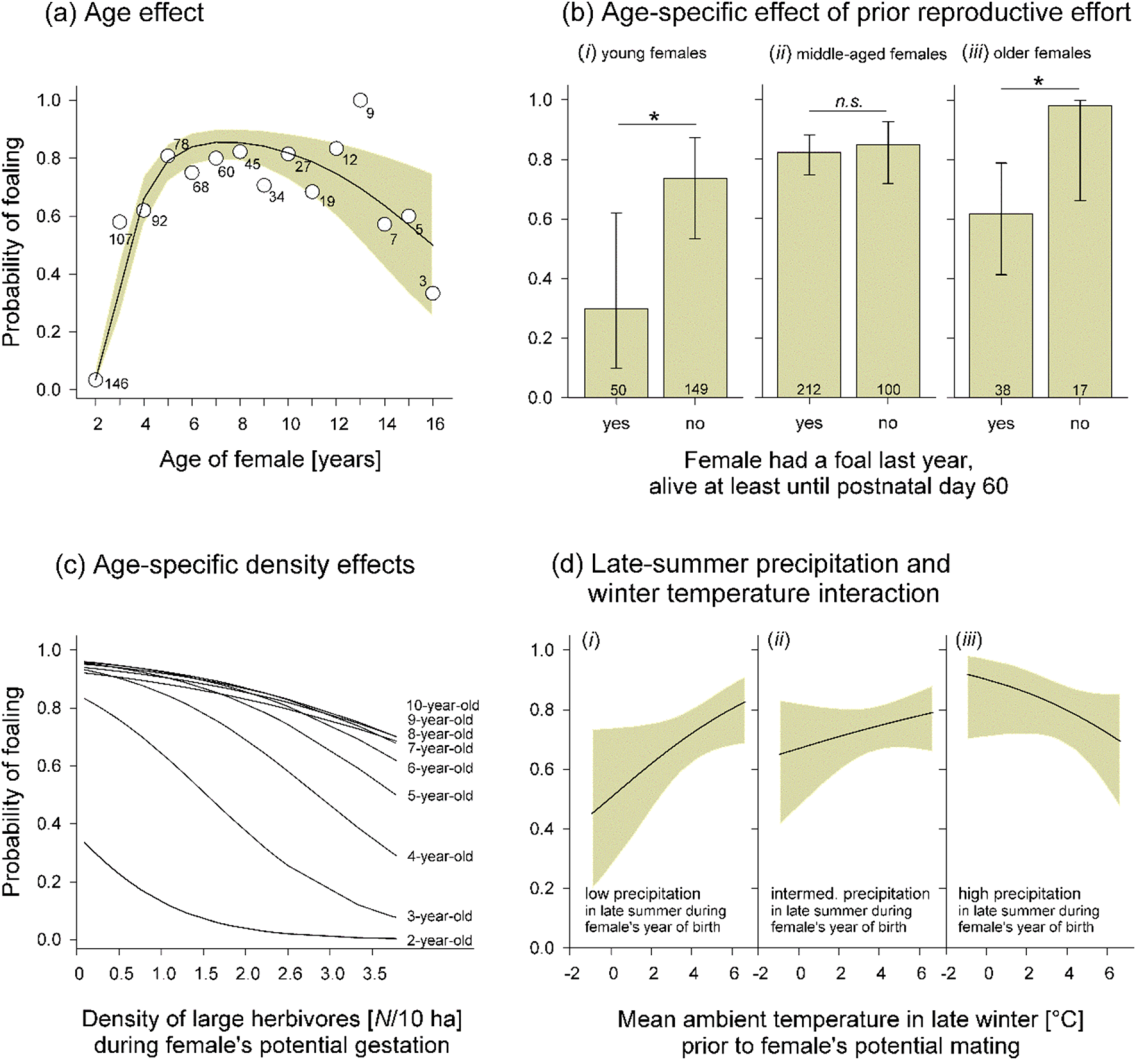
Effects of different predictors on the foaling probability of female Przewalski’s horses, aged between 2 and 16 years. Regression lines (**a**, **c**, **d**) and the bar charts (**b**) show predicted values including 95% confidence intervals (gray shading: **a**, **d**), based on estimates provided by the multifactorial model (Table 1). Data points in **a** are the average probabilities per age class; sample sizes are given beneath the circles. In **b**, model results for (i) young females (3–4 years old), (ii) middle-aged females (5–10 years old) and (iii) older females (11–16 years old) are given. The conditions in the interactive graphs (**d**) of (i) low, (ii) intermediate, and (iii) high precipitation are exemplary cases (10th, 50th and 90th percentiles) of the continuous variable *P*_*1*_ (see Table 1). All analyses were done with multifactorial GLMM for binomial data. Details on statistics for **a**, **c**, **d** are given in Table 1 (based on *n* = 712 observations from 146 individuals). For **b**, 2-year-old females were excluded from the analysis (*n* = 566 observations from 107 individuals; see Table B in Suppl. Materials)

By our analysis, we explored age-dependent changes in the probability of foaling. Therefore, in our logistic model (Table 1), we fitted female age effects by a second-order (quadratic) polynomial, which explained the associated changes in reproductive probability significantly better than a function with a simple sigmoidal shape (model comparison by likelihood ratio test: ${\chi }_{1}^{2}$ = 59.499, *p* < 0.001). This model (see significant age effect in Table 1) predicted a steep increase in the probability of reproduction in 2 to 4-year-old females, then reaching a plateau in middle-aged ones, and finally leading to a slight decrease, although with high 95% confidence interval, in old females (Fig. 2a).

#### Prior reproductive effort

As 2-year-old females inevitably had a previous reproductive investment of zero, this age class was removed from this analysis, resulting in a reduced sample size of *n* = 566 observations from 107 females. This analysis (details in Table B of Suppl. Materials) predicted a significantly lower current reproductive probability (0.710, *CI*_95%_ [0.567, 0.820]) in females which had a foal during the previous year which survived at least until postnatal day 60 (53.0% of cases), compared to females (reproductive probability: 0.870, *CI*_95%_ [0.759, 0.935]) which either did not reproduce (38.5% of cases) or had experienced an early loss of their foal during the previous season (8.5% of cases; foal mortality on average on postnatal day 6.9 ± 1.7 SE) (GLMM for binomial data: ${\chi }_{1}^{2}$ = 11.276, *β* = − 1.050 ± 0.313 SE, *p* < 0.001). All predictors and interactions among them, which were significant in the previous analysis (Table 1) were again significant (see Table B in Suppl. Materials). However, we did not find any significant interactions between the environmental variables *P*_1_, *P*_2_ and *T* and the females’ previous reproductive effort (all *p* > 0.10; Table B in Suppl. Materials). A further analysis confirmed that, as expected, the reproductive probabilities of mothers which either did not reproduce or had experienced an early loss (< postnatal day 60) of their foal during the previous season did not differ significantly (${\chi }_{1}^{2}$ = 0.204, *β* = 0.277 ± 0.613 SE, *p* = 0.651).

An additional, age-specific analysis, using age as a factor with three levels instead of as a covariate, revealed that such a negative effect of previous reproductive effort on current reproduction was only significant in the young age class of 3 to 4-year-olds (${\chi }_{1}^{2}$ = 3.955, *β* = − 1.887 ± 0.948 SE, *p* = 0.047; Fig. 2b.i) as well as in old females of 11 to 16 years (${\chi }_{1}^{2}$ = 3.999, *β* = − 3.493 ± 1.747 SE, *p* = 0.045; Fig. 2b.iii). In contrast, there was no significant effect of the previous reproductive effort in middle-aged females of 5 to 10 years (${\chi }_{1}^{2}$ = 0.219, *β* = − 0.190 ± 0.407 SE, *p* = 0.640; Fig. 2b.ii).

#### Density of large herbivores

The foaling probability of individual females depended on the density of large herbivores in an age-specific way, as evident by the statistically significant interaction between female age and density (Table 1). That is, the foaling probability showed a clear negative density dependence in young age classes, particularly visible in 3 and 4-year-olds (Fig. 2c). The slope of the negative association in 2-year-olds was comparatively lower, since females of this age class overall showed a very low probability of foaling (see also Fig. 2a). In females older than 4 years, the negative density dependence gradually disappeared with increasing age (Fig. 2c for females until an age of 11 years). More details in Fig. A in Suppl. Materials.

#### Interactive weather effects

Weather conditions significantly affected the females’ probability of parturition at multiple levels. First, this was apparent by the significant interaction between the amount of precipitation in late summer (September) during the females’ first year of life (i.e., at the foal stage, *P*_*1*_) and the average ambient temperature (*T*) in late winter (February/March) prior to the onset of the mating period (Table 1). Females, which experienced low amounts of *P*_*1*_ during their first year of life were particularly affected by negative effects of low *T* on their probability of foaling (Fig. 2d.i).

**Fig. 3:**
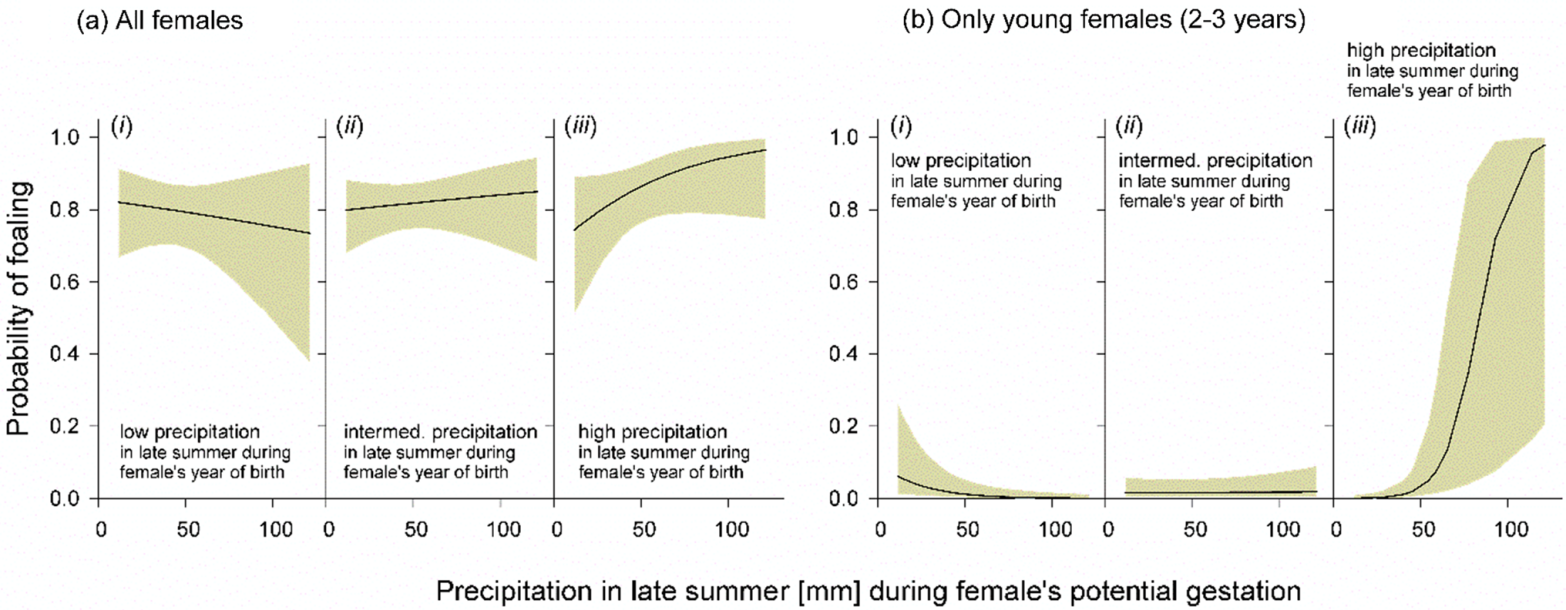
Interactive effects of late-summer precipitation (September) experienced by females’ during their first year of life and during their current (putative) gestation on the annual foaling probability of female Przewalski’s horses. Regression lines show predicted values including 95% confidence intervals (gray shading), based on estimates provided by the multifactorial model (see Table 1 and text). The conditions in the interactive graphs of (i) low, (ii) intermediate, and (iii) high precipitation are exemplary cases (10th, 50th and 90th percentiles) of the continuous variable *P*_*1*_. Analysis by GLMM for binomial data, based on data from **a** all females (*n* = 712 observations from 146 individuals) and from **b** 2-year-old and 3-year-old females (*n* = 253 observations from 146 individuals)

Second, there was a significant interaction between *P*_*1*_ and the amount of precipitation experienced in late summer during adulthood (*P*_*2*_), during the period of potential gestation (Table 1). Particularly in females which experienced more *P*_*1*_ during early life, the exposure to more *P*_*2*_ increased the probability of giving birth during the following year (Fig. 3a.iii).

An additional analysis revealed that the above-described interactive effect of *P*_*1*_ × *P*_*2*_ was also statistically significant when only considering young females of 2 and 3 years (GLMM for binomial data: ${\chi }_{1}^{2}$ = 9.636, *β* = 1.304 ± 0.420 SE, *p* = 0.002), and even showed a notably steeper slope (Fig. 3b.iii). That is, only females which experienced higher precipitation in late summer during their year of birth had a notably high probability of foaling during their 2nd and 3rd year, when late-summer precipitation during their current gestation was high. In contrast, the interaction *P*_*1*_ × *P*_*2*_ was not significant when pooling together females older than 3 years (${\chi }_{1}^{2}$ = 1.946, *β* = 0.224 ± 0.161 SE, *p* = 0.163).

### Body condition score

#### Age-specific effects of large herbivore density

**Fig. 4:**
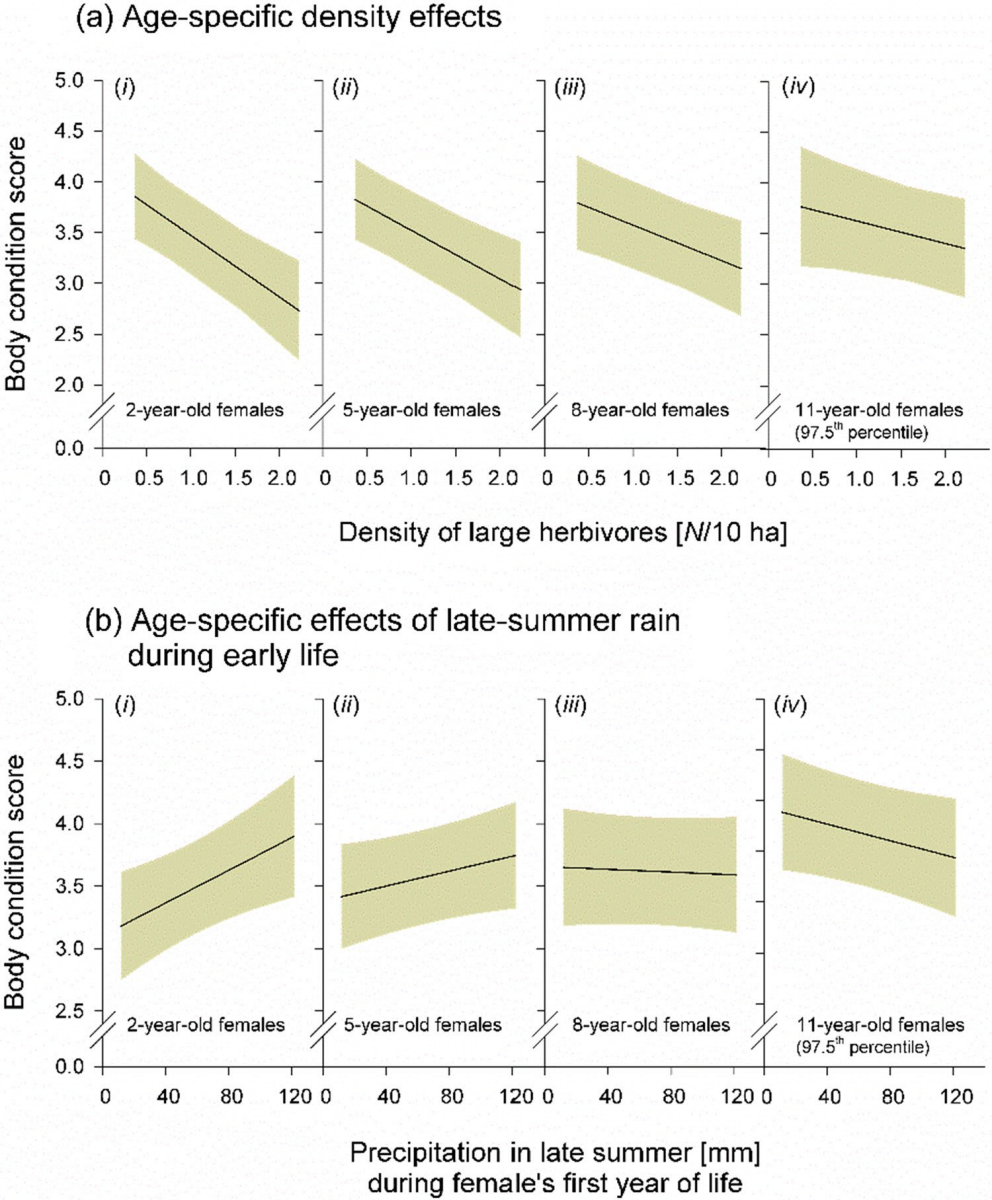
Effects of different predictors on body condition scores (after Rudman and Keiper 1991) of female Przewalski’s horses, aged between 2 and 17 years. Regression lines show predicted values including 95% confidence intervals (gray shading), based on estimates provided by the multifactorial model (Table 2). The conditions in the interactive graphs (**a**, **b**) of (i) two, (ii) five, (iii) eight and (iv) 11-year-old females are exemplary cases of the continuous variable age (*A*) (see Table 2). Analysis by LMMs with parametric bootstrapping based on body condition scores collected during from 6 years (*n* = 393 observations from 64 individuals)

The body condition of adult females decreased significantly with increasing annual large herbivore density, although in an age-dependent way as evident by the significant interaction of *D* × *A* (Table 2). That is, this negative density-dependence was particularly pronounced in young females and decreased gradually with increasing age (Fig. 4a).

**Table 2:** Effects of different predictor variables on body condition scores (after Rudman and Keiper 1991) of female Przewalski’s horses (2–17 years old)

Source of variation	*χ*^2^	*df*	*Estimates* ± *SE*	*p*
Female age *A*	0.06	1	0.027 ± 0.110	0.859
Annual large grazer density (census in late autumn) *D*	17.61	1	− 0.579 ± 0.124	**< 0.001**
Season	1.45	3		0.724
[2]			0.063 ± 0.074	
[3]			0.085 ± 0.077	
[4]			0.011 ± 0.109	
Precipitation in late summer during 1st year of life *P*_*1*_	3.59	1	0.212 ± 0.111	0.083
Precipitation in late summer last year *P*_*2*_	0.01	1	0.021 ± 0.249	0.952
*D* × *A*	5.15	1	0.140 ± 0.059	**0.039**
*P*_*1*_ × *A*	8.08	1	–0.203 ± 0.070	**0.005**
*P*_*2*_ × *A*	0.86	1	0.041 ± 0.044	0.401

Analysis by LMM with parametric bootstrapping (1000 iterations), based on *n* = 393 score values from 64 females collected during 6 years (2004–2007, 2018, 2019). All 2-way interactions between female age and the different environmental parameters were tested. Non-significant interactions (*p* > 0.05) were stepwise removed and the models were re-calculated. The proportional variance explained by the final model excluding all non-significant interactions was _marginal_ *R*^2^ = 0.196

Significant effects are highlighted in bold

Female identity and current year were used as random intercept factors. Season was a factor with 4 levels ([1] Jan–Mar; [2] Apr–Jun; [3] Jul–Sep; [4] Oct–Dec)

#### Weather effects

We found age-specific long-term effects of the amount of precipitation that females experienced in late summer during their first year of life (*P*_*1*_) on their body condition during later life, as evident by the significant interaction of *P*_*1*_ × *A* (Table 2). Young adult females of 2 to around 3–4 years which experienced higher amounts of *P*_*1*_ showed a notably increased body condition compared to females of the same age experiencing lower amounts of late-summer precipitation early in life (Fig. 4b). Although, this positive weather effect disappeared with increasing age.

Alternatively, we also tested the non-linear (second-order polynomial) effect of late-summer precipitation during early life (*P*_1_) in interaction with age. This interaction between the polynomial effect of *P*_1_ and age was also statistically significant (LMM: ${\chi }_{2}^{2}$ = 8.77, *p* = 0.018; not shown in Table 2). However, it did not explain the data significantly better than the model including the interaction *P*_1_ × *A* based on the linear effect of *P*_1_ (Likelihood ratio test: ${\chi }_{2}^{2}$ = 1.01, *p* = 0.604).

We did not detect any significant effects, including an age-specific interaction, of late-summer precipitation during the previous year (*P*_*2*_) on the body condition during the current year (Table 2).

## Discussion

### Age-specific reproductive activity

Our long-term study confirms and extends published information on reproductive parameters in the Przewalski’s horse. Parturitions peaked in mid to late May, which corresponds well to findings from other studies on free-ranging Przewalski’s horse populations (Chen et al. 2008; Dorj and Namkhai 2013). Some females of our study population had their first foal during their second year of life (more details in: Kerekes et al. 2021), although this happened only in few cases (3.4%, 5 out of 146 females included in our study). Another study on Przewalski’s horses, from a Mongolian population, found first foaling at an age of 3 years, reporting that 24.7% of females already reproduced at this age (Dorj and Namkhai 2013). Whilst such quantitative information from free-ranging Przewalski’s horses (*E. f. przewalskii*) is scarce, data on the well-studied feral horse (*E. f. caballus*) suggest a notable variation in early reproductive activity among populations and among years. Some studies reported that feral horse mares do not reproduce before reaching an age of 3 years (Seal and Plotka 1983; Garrott and Taylor 1990; Goodloe et al. 2000), whilst others found foaling in 2-year-olds with low to intermediate (Garrott et al. 1991; Linklater et al. 2004; analysis based on pregnancy rates in Grant et al. 2021), or even high rates of up to 37% (Berger 1986). Furthermore, our current study provides indications for female reproductive senescence (see reviews on mammals in: Gaillard et al. 2000; Turbill and Ruf 2010), in terms of a decreasing reproductive probability in Przewalski’s horse mares older than around 14 years, which is in accordance with studies in feral horses (Garrott et al. 1991) and in the closely related Asiatic and African wild asses (*E. hemionus*, *E. africanus*: Ibler and Fischer 2017).

### Age-dependent effects of density and weather

The prime interest of this study was to investigate possible age differences in the females’ foaling probability in response to an increased large-herbivore density and to harsh (or beneficial) weather conditions. We found consistent support that young females, as compared to middle-aged or older ones, were particularly sensitive to such environmental effects, which is in line with other studies on ungulates (review in: Gaillard et al. 2000). Extending these studies, we also found interactive environmental effects, suggesting that the interplay of weather conditions experienced during different life stages can shape female reproduction.

#### Large-herbivore density

Negative effects of population density on body condition and reproductive rates have been found in several large herbivores (e.g., Fowler 1987; Stewart et al. 2004; Bonardi et al. 2017) including in the feral horse (Garrott and Taylor 1990; Richard et al. 2014). Our study revealed pronounced negative effects of high large-herbivore density on female body condition, particularly on the body condition of the youngest female age-class considered (2-year-olds), with a gradually decreasing magnitude until the females reached an age of around 5 years (see Fig. 4a). Accordingly, negative density effects on foaling probabilities were strongest in 3- and 4-year-old females, whilst such effects were virtually absent in females older than 5 years (see Fig. 2c). Two-year-old females generally showed very low foaling rates (Garrott et al. 1991; Linklater et al. 2004), and thus negative density effects on reproduction were hardly detectable in this age class. Even though we could not directly test for a statistical association between body condition scores and foaling probability due to sample size restrictions in the former variable, we strongly suggest that the density-dependent decline in body condition of young females was the main driver of their low foaling rates under high large-herbivore densities (cf. Scorolli and López Cazorla 2000). This is further supported by a study in roe deer (*Capreolus capreolus*), showing that particularly in young females, body condition is a strong predictor of reproductive performance (Flajšman et al. 2017).

#### Interactive weather effects

Body condition scoring revealed that young females were particularly sensitive to rainy weather conditions experienced in late summer (September) during their first year of life (foal stage). Such late-summer rains typically lead to the green-up of the ground vegetation. Foals may have benefitted directly from such an increase in food availability and quality, but also indirectly via potentially positive effects of the available green pasture on mother’s lactational performance (see Morand-Fehr and Sauvant 1980 for a study in goats *Capra hircus*). Thus, females experiencing higher amounts of late-summer precipitation during early life were in a better body condition, at least during the following 1–2 years (see Fig. 4b). Such beneficial environmental conditions were also associated with the females’ foaling probability, although in an interactive manner across different life stages. Only in females, which experienced higher amounts of late-summer rain during their first year of life, a positive effect of late-summer rain during their current year of potential pregnancy was apparent. That is, only such females, at an age of 2–3 years, showed a majorly increased foaling probability during the following season when exposed to higher amounts of late-summer rain during their (potential) pregnancy (see Fig. 3b). We suggest that the increased availability of green pasture growing in late-summer and autumn may have decreased the probability that these 2- to 3-year-old females experienced pregnancy losses (Satué and Gardon 2016).

Moreover, females exposed to low amounts of late-summer rain during their first year (i.e., as foals) showed, after reaching maturity, a low probability of reproduction when experiencing harsh late-winter temperature conditions prior to the mating season. That is, in females experiencing such early-life conditions, harsh winter weather possibly limited female’s receptivity or decreased the probability of successful conceptions during the subsequent mating season. This is supported by the finding of Scheibe and Streich (2003) on another Przewalski's horse population, who have shown that harsh winter conditions, possibly via a delayed green-up of pastures, decreased the body condition of the females during the following season. Furthermore, our finding of interactive, longer-term effect of late-winter temperature on the foaling probability with a delay of 1 year extends the results of a study on feral horses in Canada, reporting that mild winters during gestation had direct, positive effects on reproductive probability and foal survival (Richard et al. 2014).

#### Potential mechanisms

What drives such a higher sensitivity to environmental conditions in young females? Adults of younger age classes, even if already reproductively active, are often not fully grown (e.g., mountain goat *Oreamnos americanus*: Houston et al. 1989; European rabbit *Oryctolagus cuniculus*: Rödel et al. 2004a), which may restrict their energy allocation to reproductive processes, in particular when food resources are limited. This might also apply to the Przewalski’s horse, as for example females of the closely related feral horse (e.g., in Australia: Csurhes et al. 2016) usually do not reach their maximum adult size until an age of around 4 years. Similar results have been obtained by studies in different breeds of domestic horses, in which females are reaching adult body size and mass at an age of around 4–5 years (Fernandes et al. 2020; Lopes Teixeira et al. 2021). A further key mechanism leading to such an age-specific sensitivity in female Przewalski’s horses, and possibly also in other group-living ungulates, could be based on the association between the females’ age and their social rank position. Female rank hierarchies are often positively correlated with age, as older females frequently occupy higher ranks whilst younger females start with subordinate positions when recruited into a social group (e.g., Rutberg 1983; Thompson 1993; Rödel et al. 2004a). Such a largely age-specific structuring of the social rank hierarchy of females, including the occurrence of agonistic behavior mainly initiated by higher ranking females towards younger, subordinate ones, has also been shown for different feral horse populations (Clutton-Brock et al. 1976; Keiper and Sambraus 1986; Heitor et al. 2006) and has been also described for two harem groups of Przewalski’s horses (Keiper and Receveur 1992). Females holding a lower social rank position frequently experience higher and even enduring levels of social stress, which can lead to an increased activation of the hypothalamic–pituitary–adrenal (HPA) axis, thus to high levels of circulating glucocorticoids (von Holst 1998; Abbott et al. 2003). Such chronically increased stress hormone concentrations can lead to immunosuppression, exert negative effects on reproductive functions and may generally make an animal more susceptible to environmental challenges (Sapolsky 1992; von Holst 1998).

In our study, an additional mechanism may have contributed to exert comparatively stronger, negative effects of large herbivore density on young females. During our 20-year study period, the population of Przewalski’s horses as well as the herd of wild cattle showed a tendency of a general increase in numbers, even though there were periods of stagnating or negative population growth (Kerekes et al. 2021). Consequently, a large proportion of younger females, which were inevitably born during the later part of the study period, had experienced higher density conditions during all their life. In turn, this was not the case for many older females, which tended to experience comparatively lower densities predominantly during their early years of life. Even though such conditions of persistent population growth over longer periods are not unusual for the dynamics of natural populations, we cannot fully exclude that the observed age-specific density effects on foaling probability may have been less pronounced in periods of a more stable or declining population density.

### Age-dependent effect of prior reproductive effort

Reproduction frequently reduces the probability that a female will reproduce during the following season (large mammals: Hamel et al. 2010b). Such a negative association indicative of fitness costs of reproduction was also apparent in our study, although in an age-specific manner. Excluding 2-year-old females from the analysis for methodological reasons (see “Methods” section), we found that young (i.e., 3- to 4-year-old) females as well as old females (> 10 years) showed a significantly lower foaling probability when they had nursed a foal for at least 60 days during the previous year (see Fig. 2b). However, there clearly were no such effects in middle-aged (5 to 10-year old, ‘prime-aged’) females, which also showed the highest foaling rates in our study (see Fig. 2a). Generally, such a pattern of higher costs of reproduction during young and/or old ages appears typical for different species of mammalian herbivores including ungulates (Clutton-Brock 1984; Proaktor et al. 2007). Age-specific reproductive costs were also apparent in a study on a feral horse population in Montana, USA, in which primiparous females, but not multiparous ones were less likely to reproduce during the following season (Garrott and Taylor 1990). A study on Soay sheep also found pronounced negative effects of previous reproductive effort on lambing probability in young (yearling) females (Regan et al. 2022). In our study, we had chosen foal survival until postnatal day 60 (i.e., at around peak lactation in domestic horses; Oftedal et al. 1983) as a proxy of mother’s prior energetic investment. Although, the vast majority, more than 90% of foals, which survived until day 60 also survived at least until the following spring. This may have potentially imposed further energetic/lactational costs to mothers during the winter, thus reinforcing the effects of previous reproduction on foaling probability during the following breeding season.

As discussed above, young female horses still need to allocate energy into their own growth until an age of around 4 years (Csurhes et al. 2016), and thus the energetic investment of young mothers into reproduction can be proportionally higher compared to older and fully grown females, possibly leading to higher reproductive fitness costs in young mothers. This hypothesis finds further support by a study on a feral horse population on an island in Nova Scotia, Canada, showing that low quality females carried the highest costs of reproduction in terms of a notably reduced foaling probability during the subsequent season (Debeffe et al. 2017). Horses, as capital breeders, can hardly compensate for a lack of energy during current reproduction via an increased food intake, which can typically lead to such delayed effects of low body condition on reproductive performance (Jönsson 1997).

At an older age, female horses in our study might have increased their relative energy allocation into current reproduction, as it can be predicted by the terminal investment hypothesis (e.g., Isaak and Johnson 2005). Such a disproportionally high reproductive investment in older mothers may have led to the observed negative effects of nursing a foal on such mothers’ subsequent foaling probability (see Fig. 2b.iii). At the proximate level, the apparently high reproductive costs in old, multiparous mothers may also be driven or reinforced by accumulating negative effects of consecutive reproductive events on females’ bioenergetic processes, constraining their reproductive abilities (cf. Zhang and Hood 2016).

## Conclusions

In summary, our study highlights a higher susceptibility of young female Przewalski’s horses to environmental challenges and to previous reproductive effort. Even though many studies on mammals have described effects of density and weather conditions on fitness-related traits such as on body condition and seasonal reproductive probability, age-dependent interactive effects of weather conditions experienced during different life stages, as we show in our study, are still rarely explored (Scorolli and López Cazorla 2000; Rödel and Dekker 2012; Richard et al. 2014). Our finding on the long-term effects of late-summer precipitation experienced by the females during their year of birth may be particularly noteworthy. Such early-life environmental effects can lead to consistent differences in traits such as body condition and reproductive performance among cohorts of individuals born during different years (Forchhammer et al. 2001; Gaillard et al. 2003), and as shown by the interactive effects in our study, may consistently affect the responses of females born during the same year to current environmental challenges, at least during younger age classes. Cohort effects may have stabilizing or even destabilizing effects on population fluctuations (Lindström and Kokko 2002), and thus knowledge of the mechanisms leading to such cohort differences may provide valuable information for the study of the dynamics and for the management of feral and Przewalski’s horse populations (Collins and Kasbohm 2017; Kerekes et al. 2021).

### Supplementary Information

Below is the link to the electronic supplementary material.

Supplementary file1 (DOCX 247 KB)

## Data Availability

Data used in the study are available from the corresponding authors upon reasonable request.
