# Supplementary material for: Age-specific effects of density and weather on body condition and birth rates in a large herbivore, the Przewalski’s horse
Source: Oecologia. 2023 Nov 16;203(3-4):435–51. doi: 10.1007/s00442-023-05477-9 (PMC10684615; doi:10.1007/s00442-023-05477-9)
Supplement: Supplementary file 1 — Supplementary file1 (DOCX 247 KB) [file 442_2023_5477_MOESM1_ESM.docx]

Oecologia

**Supplementary Materials**

Age-specific effects of density and weather on body condition and birth rates in a large herbivore, the Przewalski’s horse

Heiko G. Rödel^1,^*, Benjamin Ibler^2^, Katalin Ozogány^3,4^, Viola Kerekes^4,5,^*

^1^Laboratoire d’Ethologie Expérimentale et Comparée UR 4443 (LEEC), Université Sorbonne Paris Nord, F-93430 Villetaneuse, France

^2^Heimat-Tierpark Olderdissen (Bielefeld Zoo), Dornberger Straße 149a, D-33619 Bielefeld, Germany

^3^ HUN-REN Behavioural Ecology Research Group, University of Debrecen, Egyetem tér 1, H-4032, Debrecen, Hungary

^4^Department of Evolutionary Zoology and Human Biology, University of Debrecen, Egyetem tér 1, H-4032, Debrecen, Hungary

^5^Hortobágy National Park Directorate, Sumen utca. 2, H-4024, Debrecen, Hungary

* Correspondence

Heiko G. Rödel; *E-mail:* heiko.rodel@univ-paris13.fr

Viola Kerekes; *E-mail:* kerekes.violag@gmail.com

**Table A** Overview of the different years of study available for the analysis of foaling probabilities, as obtained from the different individuals (*n* = 146) included in our study. Note that the end of sampling data from a female can be either due to fact that the female had died, due to the translocation of the animals an adjacent enclosure or to another park, due to the fact that the study period had ended (data collection from 2000 to 2019 for this paper), or due to the female’s injection with an immunocontraceptive leading to its further exclusion from the current data analysis.

|  |  | Reasons for end of sampling | | | |
| --- | --- | --- | --- | --- | --- |
| Female age during data collection [*N* years] | *N* females | Death of female [*N*] | Translocation [*N*] | Study period ended [*N*] | Injection with contraceptive [*N*] |
| 2-years old [1 year] | 39 | 5 | 1 | 10 | 23 |
| 2-years old to 3-years old [2 years] | 15 | 2 | 1 | 9 | 3 |
| 2-years old to 4-years old [3 years] | 14 | 0 | 2 | 6 | 6 |
| 2-years old to 5-years old [4 years] | 9 | 1 | 1 | 4 | 3 |
| 2-years old to 6-years old [5 years] | 9 | 4 | 0 | 2 | 3 |
| 2-years old to 7-years old [6 years] | 15 | 1 | 0 | 4 | 10 |
| 2-years old to 8-years old [7 years] | 11 | 2 | 0 | 0 | 9 |
| 2-years old to 9-years old [8 years] | 8 | 1 | 0 | 2 | 5 |
| 2-years old to 10-years old [9 years] | 7 | 2 | 1 | 1 | 3 |
| 2-years old to 11-years old [10 years] | 5 | 0 | 0 | 0 | 5 |
| 2-years old to 12-years old [11 years] | 7 | 4 | 0 | 1 | 2 |
| 2-years old to 13-years old [12 years] | 0 | 0 | 0 | 0 | 0 |
| 2-years old to 14-years old [13 years] | 2 | 0 | 0 | 0 | 2 |
| 2-years old to 15-years old [14 years] | 2 | 0 | 0 | 0 | 2 |
| 2-years old to 16-years old [15 years] | 3 | 1 | 0 | 0 | 2 |
| **total** | **146** | **23** | **6** | **39** | **78** |

**Table B** Effects of different predictor variables on the age-specific foaling probability of female Przewalski’s horses. Analysis by GLMM for binomial data based on *n* = 566 observations from 107 females over 19 years. Non-significant interactions (*p* > 0.05) were stepwise removed and the models were re-calculated. All two-year-old females were excluded from analysis; see results text for details. The proportional variance explained by the final model excluding all non-significant interactions was _marginal_ *R*^2^ = 0.203.

| Source of variation | *χ*^2^ | *df* | *Estimates ± SE* | *p* |
| --- | --- | --- | --- | --- |
| Female age (2^nd^ order polynomial effect) *A* | 28.795 | 2 | –12.603 ± 3.236 | **< 0.001** |
| Large grazer density after potential conception *D* | 18.849 | 1 | –1.166 ± 0.268 | **< 0.001** |
| Previous reproductive effort *R* | 11.276 | 1 | –1.050 ± 0.313 | **< 0.001** |
| Precipitation in late summer during 1^st^ year of life *P*_1_ | 1.113 | 1 | 0.156 ± 0.148 | 0.291 |
| Precipitation in late summer after potential conception *P*_2_ | 0.101 | 1 | 0.073 ± 0.230 | 0.750 |
| Temperature in late winter prior to potential conception *T* | 0.767 | 1 | 0.217 ± 0.248 | 0.381 |
| *D* × *A* | 4.260 | 1 | 0.398 ± 0.193 | **0.039** |
| *P*_1_ × *A* | 0.752 | 1 | –0.168 ± 0.194 | 0.385 |
| *P*_2_ × *A* | 0.354 | 1 | 0.113 ± 0.189 | 0.552 |
| *T* × *A* | 1.102 | 1 | –0.224 ± 0.214 | 0.294 |
| *R* × *D* | 2.321 | 1 | 0.487 ± 0.320 | 0.128 |
| *R* × *P*_1_ | 0.294 | 1 | 0.131 ± 0.241 | 0.588 |
| *R* × *P_2_* | 0.333 | 1 | 0.157 ± 0.272 | 0.564 |
| *R* × *T* | 1.968 | 1 | –0.428 ± 0.305 | 0.160 |
| *P*_1_ × *P*_2_ | 4.530 | 1 | 0.358 ± 0.168 | **0.033** |
| *P*_1_ × *T* | 4.589 | 1 | –0.281 ± 0.131 | **0.032** |
| *P*_2_ × *T* | 0.327 | 1 | 0.225 ± 0.393 | 0.567 |
| *P*_1_ × *D* | 2.243 | 1 | –0.281 ± 0.188 | 0.134 |
| *P*_2_ × *D* | 2.173 | 1 | –0.464 ± 0.314 | 0.140 |
| *T* × *D* | 0.738 | 1 | 0.213 ± 0.248 | 0.390 |

*Notes:* Female identity and current year were used as random intercept factors.


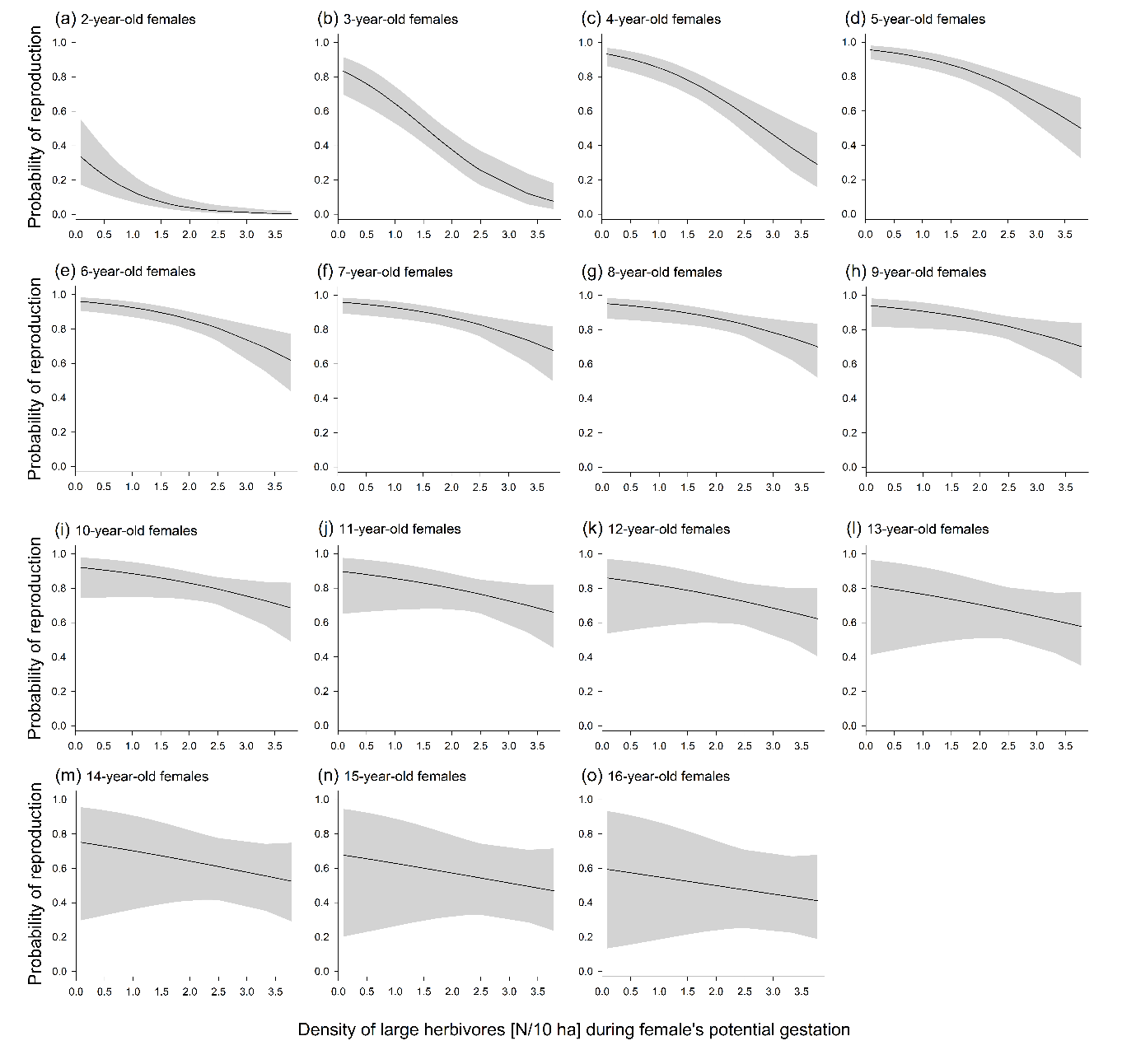


**Fig. A** Age-specific effects of the density of large herbivores (numbers of Przewalski’s horses and of cattle per 10 hectares) during autumn/winter, i.e. during the females’ potential gestation period on females’ foaling probability. Regression lines show predicted values including 95% confidence intervals, based on estimates provided by the statistically significant interaction between large herbivore density and female age (see Table 1). Analysis by GLMM for binomial data, based on data from 20 years (*N* = 705 observations from 146 mothers). Note that in this analysis, age was used as a covariate.
